# Tramadol misuse in treatment-seeking adolescents and young adults with problematic substance use – Prediction of treatment retention

**DOI:** 10.1016/j.abrep.2022.100446

**Published:** 2022-07-11

**Authors:** Eleonora Almér Herrnsdorf, Alexander Holmstedt, Anders Håkansson

**Affiliations:** a Lund University, Faculty of Medicine, Department of Clinical Sciences Lund, Psychiatry, Lund, Sweden; b Malmö Addiction Center, Region Skåne, Malmö, Sweden

**Keywords:** Tramadol, Adolescents, Non-medical prescription use of opioids, Retention, Substance use disorder

## Abstract

Non-medical prescription use of opioids (NMPUO) is a public health concern worldwide. Recently, tramadol misuse is increasing, but the systematic research of misuse of this specific opioid is limited. This study set out to assess the relationship between tramadol use and completion of treatment for substance use among adolescents and adults ≤ 25 years in an outpatient clinical setting. A retrospective cohort study of treatment outcome, expressed as “completion” or “non-completion” of treatment, was conducted in treatment-seeking adolescents with problematic substance use (n = 335). Data was extracted from Ung-DOK interviews, a semi-structured assessment instrument designed for adolescents with substance abuse. The study included all treatment-seeking patients at an out-patient facility in 2014–2017. A total of 26% (n = 88) were tramadol users (life-time prevalence). Twenty percent (n = 66) of all treatments were non-completed. Tramadol users were significantly more likely than non-users to drop out of treatment (35% vs 15%, p < 0.001). In multivariate logistic regression, tramadol use and age 18 and above were factors significantly associated with non-completion. Tramadol use was statistically significantly associated with non-completion of treatment. Further research addressing treatment needs and treatment completion among tramadol users is needed.

## Highlights

• Misuse of tramadol is increasingly highlighted as a problem in adolescents and adults.
• In treatment for substance use in the young, tramadol use increased the risk of treatment drop-out.
• Tramadol misuse, hitherto little examined, requires further clinical research.

## 1 Introduction

Non-medical prescription use of opioids (NMPUO) is a growing public health concern worldwide. For example, in 2017, the prevalence of NMPUO was 4% among the North American population aged 15–64, where “an alarming increase in the number of fatal and non-fatal opioid overdose cases reported” has been seen during the last decades (United Nations Office on Drugs and Crime, 2019). In the World Drug Report of 2019, the misuse of tramadol is referred to as “the other opioid crisis”. Reports of increased tramadol misuse emerge from many sub-regions worldwide, particularly West, Central and North Africa and also the Middle East, other parts of Asia, Europe and North America (Iravani et al., 2010, Bassiony et al., 2015, United Nations Office on Drugs and Crime, 2019).

Sweden has seen an increase of non-medical prescription use of tramadol, especially among adolescents. The city of Malmö, in the south of Sweden, appears as a particularly vulnerable area (Richert, & Johnson, 2013). The amount of non-medical prescription drugs seized by Swedish police authorities has increased over the last decade, with tramadol being the second most common seized illicit drug (CAN, 2019). Tramadol is controlled in Sweden since 2007 (Swedish Medical Products Agency, 2006), and the number of individuals being medically prescribed opioids has been constant between 2006 and 2015, pointing towards “non-iatrogenic” sources of the increased availability on the illicit market (Bäckryd, Heilig, & Hoffmann, 2017).

Tramadol is a centrally active analgesic indicated for treating moderate to severe pain (United Nations Office on Drugs and Crime, 2019). Approved in 1995 by the Food and Drug Administration (FDA), it was initially launched as the only uncontrolled opioid available, due to its apprehended low risk for adverse effects and addiction (Miotto et al., 2017). Tramadol produces analgesia in a multimodal fashion via opioid as well as norepinephrine (NA) and serotonin (5HT) systems. In regard to this complexity, also the adverse effects show a multifaceted pattern (Grond, & Sablotzki, 2004). The predominant analgesic effect of tramadol is mediated by its active metabolite, O-desmethyltramadol (M1), which has around 300 times higher affinity for the my-receptor than the parent compound (Gillen, Haurand, Kobelt, & Wnendt, 2000).

To date, several lines of evidence support that tramadol can cause seizures (Spiller et al., 1997, Shadnia et al., 2008), and that seizures are more likely to occur in patients exceeding recommended doses of tramadol, but also occur in patients receiving the substance within a recommended dose range (Grond, & Sablotzki, 2004). Furthermore, there have been reports of serotonergic syndrome occurring in combination with other serotonergic drugs (Kaye, 2015), central nervous system depression, respiratory depression and death (Miotto et al., 2017, Randall and Crane, 2014). Similar to other opioids such as morphine and heroin, tramadol use may be associated with tolerance, dependence and addiction liability (Zhang and Liu, 2013, Tjäderborn et al., 2009, Zacny, 2005).

While there is a growing body of literature that recognizes tramadol as a major health problem, the systematic research on motivation and patterns of misuse of tramadol is hitherto limited. According to the World drug report of 2018, tramadol misuse appears to be deviating from that of other opioids. It attracts novel groups in society, less commonly using other opioids, and the mood elevating property of tramadol has been proposed an explanation to this (United Nations Office on Drugs and Crime, 2018). Winstock et al. (Winstock, Borschmann, & Bell, 2014) conducted an online survey that revealed usage of tramadol not only for pain relief (75%), but for reasons such as to relax (31%), get high (25%), to relieve boredom (16%) and relieve anxiety (10%). Similarly, an American study assessing drug behavior among high school seniors, identified five motivational subtypes related to NMPUO: “experiment, relax, get high, pain relief, and affect regulation” (McCabe, & Cranford, 2012). Furthermore, studies suggest a contributing factor to the high level of misuse of tramadol is the perception among users that it is “safe”, attributed to it being a pharmaceutical drug (Barati, 2014). Various studies have also pointed towards increasing use of tramadol as an off-label remedy to premature ejaculation, and concern is being raised regarding media targeting of young male and promotion by online drug stores (Fawzi, 2011, Ibrahim et al., 2017).

In a recent Swedish study analyzing hair-samples, Olsson et al. (Olsson, Öjehagen, Brådvik, Kronstrand, & Håkansson, 2017) found tramadol to be the predominating opioid misused among treatment-seeking adolescents at the outpatient clinic Maria Malmö (31% tested positive of tramadol). Furthermore, polydrug use (use of multiple substances within a specific period of time) was more frequent among the tramadol users than in other substance users. This finding is consistent with a growing body of literature showing a polydrug pattern associated with tramadol (Bassiony et al., 2018, Nazarzadeh et al., 2014), and polydrug use is known to be associated with a more severe clinical presentation and poorer treatment outcome (Williamson, Darke, Ross, & Teesson, 2006).

Thus, tramadol presents a relatively novel drug use pattern in some geographical settings, such as the one studied here. In 2007, the journal of the Swedish Pharmaceteutical Association reported that tramadol, at that time still a non-controlled drug, was increasingly misused by adolescents, and anecdotal reports claimed that tramadol use may be particularly difficult to withdraw from (Läkemedelsvärlden, 2007). From a treatment perspective, in substance use disorders in general, premature dropout of patients is common in treatment. Dropout rates range from 23 to 50% in outpatient treatment for substance use disorder (Brorson, Arnevik, Rand-Hendriksen, & Duckert, 2013), and a considerable body of research has searched for risk factors for non-completion of substance use treatment (Brorson et al., 2013). According to Brorson et al. (2013), a total of five out of 14 studies that were focusing on opiates, found significant associations with drop-out: four studies concluded opiates increased the risk of drop-out, and one study concluded that opiates were unrelated to drop-out. In reviewing literature, the relationship of tramadol as the primary substance and drop-out of treatment is hitherto understudied.

Given the novelty of this substance in drug use patterns, its somewhat atypical effects in comparison to other opioids, possibly presenting new challenges in opioid withdrawal and treatment, there is reason to study how tramadol use may affect dropout from out-patient treatment for drug use problems in young patients. Thus, the present study in a clinical setting in Malmö, Sweden, aimed to examine whether a poor treatment outcome, defined as the non-completion of treatment, was more common in tramadol users than in other substance-using adolescents and young adults.

## 2 Methods

The Maria Malmö outpatient clinic, in Malmö, Sweden, is an outpatient clinic aimed at adolescents and adults up to 25 years old with substance use disorders. The clinic has an uptake area of approximately 340,000 habitants and is administered by psychiatric health care centers (Malmö Addiction Center and Department of Child and Adolescent Psychiatry, Skåne) and the social resource management of Malmö City.

A baseline interview, using the interview instrument Ung-DOK, is mandatory and performed on all clients upon enrolment at Maria Malmö treatment center. After mapping the care need, clients are offered individually adapted psychosocial and medical treatment. This may consist of motivational interviewing (MI), cognitive behavioral therapy (CBT), relapse prevention (RP), family therapy/support, behavioral self-control training (BSCT), manual based session programs for young chronic cannabis users (HAP-CPU), parent/relative support and/or education, medical assessment, ambulatory withdrawal treatment, drug testing, and pharmacological treatment. The frequency and length of contact is individual, but most commonly consists of 1–2 sessions weekly for 3–6 months. Follow-up is offered three months after end of contact.

At the end of a treatment, an Ung-DOK follow-up is performed (for description of study instruments, see below). In case the patient has dropped out from treatment, this is noted in the follow-up instrument, from which the data on treatment completion vs non-completion in the present study is derived.

Retrospective data on the subjects included (from the baseline interviews and the follow-up data) was collected in anonymous and confidential form, from the Ung-DOK database (administered by the Linnaeus University, Växjö) which collects data continuously as part of a national project documenting substance use among treatment-seeking adolescents on group-level for quality and clinical research purposes (Holmstedt et al., 2020). The present study was approved by the Regional Ethics Committee in Lund (file number 2018/165). Based on its design as a retrospective clinical documentation study, the study did not require information to patients and informed consent procedures.

### 2.1 Study participants

The present study included all patients with available baseline data from 1st of January 2014 to 31st of December 2017. This sample included a total of 639 patients. For patients ending treatment during this period, there was also an Ung-DOK follow-up interview completed, and paired to the baseline interview via an ID. In this study, 109 baseline interviews were identified as the second or third of the same individual, and hence excluded. Two were incomplete and four could not be paired with an Ung-DOK follow-up interview and were therefore also excluded. Furthermore, an inclusion criterion for this study was that subjects had reported use of any illicit drug (that is, not only alcohol) in their baseline interview, excluding 189 subjects. This resulted in 335 Ung-DOK interviews being included in this study.

### 2.2 Measures

Variables assessed were derived from the interview instrument Ung-DOK (“Young-DOK”), the adolescent version of the instrument DOK (Swedish abbreviation for “Documentation and evaluation regarding treatment of alcohol and drug abusers”) (Anderberg, & Dahlberg, 2010). The overall structure of DOK is similar to that of ASI (Addiction Severity Index), a well- established multi-dimensional interview instrument, designed to measure, characterize and quantify the severity of social problems, health and substance use in individuals with problematic use of alcohol or other substances (McLellan, Cacciola, Alterman, Rikoon, & Carise, 2006). Ung-DOK is a semi-structured interview method designed specifically for adolescents with problematic use of alcohol and other drugs, where the subject and a therapist go through the questions together. The interview contains questions addressing sociodemographic background, housing and financial support, occupational status, substance use (alcohol, drugs, tobacco), treatment history, criminality, childhood, exposure to violence, family and relationships, physical and mental health (Anderberg, & Dahlberg, 2010).

In the Ung-DOK baseline interview the primary drug used is defined, and if relevant, what secondary drugs may constitute a problem for the subject. The drugs specified as problem drugs are “alcohol, cannabis, amphetamine, cocaine, ecstasy, LSD, heroin, methadone, buprenorphine, GHB, spice (synthetic cannabinoids), solvents, benzodiazepines, opiates and other sedatives, anabolic and androgenic steroids” and “others”. Thus, as the Ung-DOK interview is primarily oriented towards the assessment of clinical treatment needs, the definition of “users” and “non-users” of a specific substance relates to whether there has ever been a use causing any problem.

Study items included in the present study, as potential correlates of treatment completion vs dropout, include the following: history of mental health and substance use disorder treatment (history of any contact with general psychiatry and/or child and adolescent psychiatry, history of previous contact with the present addiction unit, contact with social services, history of residential treatment, history of compulsory treatment for adolescents, history of drug use or alcohol use disorder treatment), lifetime history of mental health problems (depressive symptoms, anxiety, suicidal ideation), regular tobacco use, lifetime history of problematic substance use (cannabis, amphetamine, cocaine, ecstasy, LSD, heroin, methadone, buprenorphine, GHB, spice, solvents, benzodiazepines, opioids, tramadol, anabolic and androgenic steroids), history of criminal justice contact, history of conviction for crime, gender, and age (the latter dichotomized into 18 years or older vs younger than 18 years). All varibles included were dichotomous items derived from the Ung-DOK interview, i.e. no continuous variables or other assessment scales were used.

The Ung-DOK follow-up interview consists of items evaluating the progress and results of treatment at its end, including items regarding change in status of mental health and change in status of the subjects’ situation with narcotics and medication. One item specifies weather the ending of the treatment was “planned”, “completed through referral to another instance of care” or “unplanned”. Unplanned ending is here understood as “dropping out” of treatment before the medical treatment was completed or quitting treatment prior to its official ending, without announcing the therapist. Unplanned ending will further be referred to as “non-completion of treatment”, as oppose to “completion of treatment”.

### 2.3 Statistical methods

All statistical analyses were carried out in the software SPSS (IBM SPSS statistics version 25). P values<0.05 were considered significant. Chi-square tests (and Fisher’s exact test in case of too small group sizes) was used to examine potential statistically significant differences between treatment completers and others, with respect to gender, age groups (<18 years old vs ≥ 18 years old), tramadol use and use of each of the substances assessed in the Ung-DOK, history of depression, anxiety and suicidal ideation, and history of each type of substance use treatment, psychiatric treatment, social services treatment, and criminal charges. All variables associated with treatment completion (p < 0.05) in these binary analyses, along with gender and age for control, were entered in a logistic regression analysis, with non-completion of treatment as dependent variable. The results were presented as odds ratios (95% confidence interval).

## 3 Results

A majority of participants (74 percent, n = 249) were men. A majority (60 percent, n = 201) were below 18 years of age. The age varied between 13 and 24 years, with a median of 17 (IQR 16, 20) and a mean age of 18 (SD = 2.6). Eighty-eight (26%) of the subjects stated any problematic tramadol use in their drug history, whereof 29 subjects stated tramadol as their primary drug. A total of 268 (80%) of treatments at Maria Malmö were completed (234 endings were planned; 34 ended through referral to another care instance). Sixty-six (20%) of the treatments were non-completed.

Tramadol users (p < 0.001), ecstasy users (p = 0.02) and cocaine users (p = 0.04) were significantly less likely than non-users to complete treatment. Also, treatment completers were less likely to report criminal conviction (p = 0.02), and less likely to report a history of depressive symptoms (p < 0.05) or suicidal ideation (p < 0.05). Other variables tested were not significantly associated with treatment completion (Table 1). When significant variables were entered in a multivariate logistic regression, along with gender and age, tramadol use remained significantly negatively associated with treatment completion, along with age above 18 years being negatively associated with completion (Table 2). Given the overwhelming majority of respondents who were cannabis users, a sub-analysis was done comparing treatment completion in combined cannabis and tramadol users, compared to cannabis users without tramadol use. Here, treatment completers were significantly less likely (p < 0.01) to be combined cannabis/tramadol users (22 percent, n = 58, vs 41 percent, n = 27).

**Table 1:** Analysis of variables associated with treatment completion among 335 substance-using adolescents and young adults. Chi-square test comparing treatment completers and non-completers.

	**Completers, n (%)**	**Non-completers, n (%)**	***p* value**
Cannabis use^1^	259 (97)	63 (95)	0.42
Amphetamine use	26 (10)	9 (14)	0.36
Ecstasy use	41 (15)	18 (27)	0.02*
Cocaine use	53 (20)	21 (32)	0.04*
Heroin use	6 (2)	2 (3)	0.66
Methadone use	0 (0)	0 (0)	1.00
Buprenorphine use	5 (2)	3 (5)	0.20
LSD use	15 (6)	6 (9)	0.30
GHB use	1 (0)	1 (2)	0.36
Spice use	38 (14)	14 (21)	0.17
Solvents use	3 (1)	0 (0)	1.00
Benzodiazepine use	29 (11)	12 (18)	0.11
Opioids	28 (11)	10 (15)	0.29
Anabolic-androgenic steroid use	1 (0)	0 (0)	1.00
Tramadol use	58 (22)	30 (45)	<0.001*
Tobacco use regular	196 (74)	53 (80)	0.31
Female gender	66 (25)	18 (27)	0.68
Age 18 years or above	89 (33)	43 (65)	<0.001*
Social services contact ever	120 (45)	28 (44)	0.82
Ever treated in residential treatment	48 (19)	14 (21)	0.63
Ever treated in compulsory care for adolescents	8 (3)	3 (5)	0.54
Any previous contact at the present unit	25 (9)	6 (9)	0.94
Any contact with general psychiatry / child and adolescent psychiatry	71 (27)	11 (17)	0.09
Ever treated for drug problems	35 (13)	14 (21)	0.10
Ever treated for alcohol problems	5 (2)	0 (0)	0.26
Ever depressed	173 (66)	51 (79)	<0.05*
Ever anxiety	183 (69)	49 (75)	0.34
Suicidal ideation ever	83 (32)	29 (45)	<0.05*
Sentenced for crime	59 (24)	22 (38)	0.02*
History of psychiatric health care	121 (46)	29 (45)	0.88

1 Fisher’s exact test used instead of chi-square test.

**Table 2:** Multivariate logistic regression analysis of variables predicting treatment completion (subjects with full data, n = 305).

	**p-value**	**OR (95% confidence interval)**
Tramadol use	<0.01	0.40 (0.21–0.76)
Gender	0.71	1.15 (0.55–2.41)
Age (18 years or older)	<0.01	0.36 (0.18–0.74)
History of depression	0.59	0.81 (0.37–1.76)
History of suicidal ideation	0.45	0.77 (0.38–1.54)
Sentenced with crime	0.74	0.89 (0.44–1.78)
Cocaine use	0.92	1.04 (0.48–2.24)
Ecstasy use	0.71	0.85 (0.37–1.96)

## 4 Discussion

The main finding of this study was the statistically significant association between tramadol use and non-completion of treatment. No other specific substance was associated with treatment completion in controlled analyses. Another important finding was that age above 18 was significantly associated with treatment dropout. In treatment of substance use problems in adolescents and young adults, while completers and non-completers did not differ on most items related to substance use or treatment history, after controlling for other factors which displayed some significance difference between groups, completion remained associated only with tramadol use and older age. Thus, the use of tramadol specifically appears to play an important role in dropout from treatment of young drug users. The findings call for further research and clinical attention to tramadol use as particularly problematic and possibly linked to a more problemtic course in treatment than several other drugs.

Dropout from treatment constitutes a major challenge in the treatment of substance use disorders generally. In a large-scale systematic review, Brorson et al. (2013) analyzed studies on premature attrition of treatment for substance use, searching for suggested risk factors that would be consistent across different study designs, samples and measurement methods. They found that cognitive deficits (specifically those related to an impaired prefrontal cortex), low treatment alliance, personality disorder, and younger age were risk factors consistently associated with non-completion. Previous studies investigating correlations between primary substance and early dropout have observed inconsistent results. Given the association of tramadol use with treatment dropout in the present study, while such findings were not seen for other substances and few differences generally emerged between completers and non-completers, further research should include tramadol in the assessment of dropout risk in substance use disorder treatment in the young.

It is difficult to explain the discrepancy in non-completion between tramadol users and non-users in our study, but it might be related to the dual pharmacological features of tramadol leading to a complex role of this particular pattern of substance misuse. While the possible interference of other risk factors, such as the ones suggested above by Brorson et al. cannot be ruled out, the current study raises the possibility that tramadol use may be a risk factor for non-completion of treatment among adolescents. According to prior studies, premature drop out is associated with high risk for poor health-related and social outcome (Dalsbø et al., 2010).

Contrary to expectations, the current study found that younger age (under 18 years) was significantly associated with being more likely to complete treatment. Conversely, as mentioned above, earlier studies have found that younger age is a small but consistent risk factor for drop-out of substance abuse treatment (Brorson et al., 2013). This has been attributed to a general tendency among adolescents towards impulsivity and risk taking, in comparison with adults (Arnett, 1992), in line with a lower level of cognitive and behavior control exhibited in the young (Thompson-Schill, Ramscar, & Chrysikou, 2009). Interpreting the current study results is dependent on where we put the cut-off between “old” and “young”. In a broader research context on substance use including adults, the subjects in our study were all young (age 13 to 24). One possible explanation to the findings in our study could be that being under 18 years old is related to having more protection factors against drop-out of treatment. Adolescents under 18 years old are to a greater extent still in school and/or live at home, which may provide external supervision and support. Moreover, being between 18 and 25 could be related to having more risk factors for dropping out. It has been shown that individuals aged 18 to 25, is a particularly vulnerable group among substance users. According to a 2017 report from the Swedish Health and Social Care Inspectorate, substance users aged 18 to 25 years were, compared with other age groups among substance users, more likely to have psychiatric comorbidity and less likely to have the security of having a job and a residence. Furthermore, they have not yet experienced the negative consequences of substance misuse that could provide a motivation for quitting (Öström, 2017).

According to expectations, this study found that tramadol-using participants had a high frequency of being involved in crime (either ever been arrested/carried by police or convicted). This finding is consistent with that of Olsson et al. (2017) who found the young tramadol users at Maria Malmö to have a higher frequency of being convicted of crime compared with other young drug users. Prior studies have found substance use to be more prevalent among populations of criminal justice than in a general population (Teplin, Abram, McClelland, Dulcan, & Mericle, 2002) and juvenile drug use is a predictor of engaging subsequently in a criminal career (DeLisi, Angton, Behnken, & Kusow, 2015).

The subjects in this study reported a high frequency of mental health issues. A little less than half the subjects in the cohort had been in contact with psychiatric care. These findings corroborate prior studies showing increased frequency of mental health issues among adolescent substance users compared with non-substance-using peers (Deas, & Brown, 2006). Contrary to expectations however, tramadol users in our study were neither more likely to have had mental health issues nor psychiatric care than other treatment-seeking adolescents. In reviewing literature on the relationship between tramadol misuse and psychiatric comorbidity, Bassiony et al. (Bassiony, Youssif, Hussein, & Saeed, 2016) found that patients with opioid use disorders related to tramadol had significantly higher prevalence of psychiatric comorbidity, when compared with a non– substance using control group (49% vs 24%). Another recent study found that concomitant use of tramadol and cannabis was common among a population presented with first episode psychosis, indicating a possible correlation between tramadol use and psychiatric disorders (Taha, Taalab, Abo-Elez, & Eldakroory, 2019). Here, when controlling for other variables, mental health problems were not significantly associated with treatment dropout, although some differences appeared in the non-adjusted, binary analyses. Furthermore, considering the non-extensive and self-reported design of the item assessing mental health in the Ung-DOK instrument (respondents were simply asked to state if they had *ever experienced anxiety or depression*), the results of our study regarding tramadol use and psychiatric comorbidity should be interpreted with caution.

Considering that misuse of tramadol is a growing public health concern internationally, with possibly fatal consequences for the individual, and vast costs for society, identification of specific features of tramadol misuse is essential. Increased systematic knowledge of consumption pattern, purpose of use and adverse effects of tramadol will assist current caregivers in tailoring clinical treatment. It will also facilitate the implementation of interprofessional work in order to meet specific care needs for tramadol-users with for example psychiatric comorbidity, weight loss or sexual dysfunction. Moreover, the knowledge should be used to improve assessment tools for misuse of tramadol among adolescents at various stages of abuse. This may increase chances of early intervention in order to minimize negative consequences of tramadol abuse and addiction.

More research on larger study samples is needed. To develop a broader picture of tramadol misuse among adolescents, additional studies are needed that bring together and compare different stages of abuse, including “recreational users”, “treatment-seekers” and “non– treatment-seekers”. Moreover, this study was cross-sectional and there is a need for longitudinal studies to explore clinical development and outcome over time in adolescents using tramadol. This includes finding predictors for further substance misuse of tramadol and/or other illicit drugs. A suggested study would be to investigate the prevalence of tramadol misuse in Ung-DOK records of adults currently enrolled in substance abuse treatment, e.g. opioid maintenance treatment (OMT). Considering the particular features of tramadol misuse, another suggested work would be to introduce a specific item focusing on tramadol use in current/new assessment tools for substance use.

There are however limitations to this study. Generalizability may have been affected by small study samples and a singular treatment center setting. Moreover, this study relies exclusively on self-report of substance use, a method depending on retrospective recall, and participants may misreport current drug use. The results of the section in this study considering change in outcome of treatment should also be interpreted with caution due to limitations of the assessment tool in examining this matter.

In addition, it should be borne in mind that a number of factors may affect the likelihood of drop-out from treatment, including both underlying risk factors such as socio-demographic and personality variables, as well as dynamic risk factors such as events occurring to the individual during treatment. One limitation of the present study is that being a real-world observational clinical study, it cannot hold potential risk factors constant. Thus, tramadol users and non-users may differ in their risk factor profile from baseline, which is adjusted for – for only to a certain extent – in the multivariate analysis of the study. Thus, future research in the area should include more in-depth measures of socio-demographic baseline and personality characteristics. Somewhat surprisingly, treatmetn completers and non-completers differed only on a few items, while a substantial number of items describing problem severity or previous treatment needs were similar between the groups. A relatively high number of variables tested may confer a certain risk of mass significance, i.e. the appearance of random associations without real significance. Here, however, few significant differences were seen, and only significant variables were further entered into the regression model where these items were controlled for one another. The reporting of a number of non-significant variables strengthen the picture that patients dropping out from treatment differ from completers on only a limited number of items, one of which was the use of tramadol.

## 5 Conclusions

In treatment of adolescent drug use, tramadol use, in contrast to other substances used by substance-using adolescents, may present a particularly large challenge to treatment completion. In addition, within the group of young patients, older age appears to be associated with non-completion of treatment. Adolescent and young adult tramadol users may constitute a particularly vulnerable group with specific care needs and a higher risk of dropping out of treatment. Given the relative novelty of tramadol in the drug scene in the present setting and others, further research is needed in tramadol misuse and the challenges it may present in treatment.

## CRediT authorship contribution statement

**Eleonora Almér Herrnsdorf:** Conceptualization, Data curation, Formal analysis, Investigation, Methodology, Validation, Visualization, Writing – original draft. **Alexander Holmstedt:** Conceptualization, Data curation, Funding acquisition, Investigation, Methodology, Resources, Writing – review & editing. **Anders Håkansson:** Conceptualization, Data curation, Funding acquisition, Investigation, Methodology, Project administration, Resources, Software, Supervision, Validation, Writing – review & editing.

## Declaration of Competing Interest

The authors declare that they have no known competing financial interests or personal relationships that could have appeared to influence the work reported in this paper.
